# Impacts of wild blueberries on weight regulation, appetite, and cardiometabolic biomarkers of adults: A randomized clinical trial

**DOI:** 10.1016/j.metop.2026.100514

**Published:** 2026-10-22

**Authors:** Mark Kern, Parker Contreras, Emily Hickey, Sarah Parsons Lee, Jenna Mueller, Shirin Hooshmand

**Affiliations:** School of Exercise and Nutritional Sciences, San Diego State University, San Diego, CA, USA

**Keywords:** Anthocyanin, Polyphenols, Food matrix, Satiety, Obesity, Yogurt

## Abstract

Blueberries are rich in polyphenols and other bioactive compounds that may influence appetite regulation, weight management, and cardiometabolic health. The effects of daily wild blueberry consumption on appetite, body weight (primary long-term phase endpoint) and composition, and cardiometabolic biomarkers were evaluated during an energy-restricted weight loss intervention. In a randomized, single-blind, parallel-arm trial, 38 adults (BMI 25–40 kg/m^2^) were randomly assigned to consume daily a 170 kcal yogurt parfait made with either 1 cup of wild blueberries (n = 18 completers) or with an isocaloric artificially-flavored, sugar-sweetened blueberry syrup (n = 18 completers) for 8 weeks within a structured weight loss program. Acute postprandial (2 h) appetite ratings and hormonal responses (glucose, insulin, ghrelin, pancreatic polypeptide) were assessed at baseline as primary acute phase endpoints. Secondary outcomes included changes in body composition, blood lipids, glucose, insulin, C-reactive protein, blood pressure, food cravings and self-reported appetite and physical activity. Both groups exhibited significant weight loss (1.1 ± 1.8 kg for blueberry vs. 1.3 ± 1.3 kg for syrup) over time (p < 0.05) with no differences between treatments, despite a tendency for blueberries to enhance cravings control compared to syrup (p = 0.059). Main effects of time for reduced lean and fat masses and body water were detected, but post-hoc analyses revealed significant (p < 0.05) differences within syrup consumers only. No significant between-group differences were observed for cardiometabolic biomarkers. Insulin tended (p = 0.055 for main effect of time) to decrease but with no differences between groups. Acutely, both groups exhibited similar changes in appetite ratings and hormones over time. Consumption of intact wild blueberries within a yogurt-based snack did not impact acute appetitive responses or longer-term weight loss and cardiometabolic outcomes compared to a refined syrup-sweetened control yogurt during 8 weeks of energy restriction despite a tendency to improve cravings control.

ClinicalTrials.gov ID: NCT05736432.

## 1 Introduction

Wild blueberries are a rich source of numerous nutrients and bioactive compounds that may impact weight and appetite regulation as well as cardiovascular disease risk factors; however, some of these potential effects have not been well-studied for either wild blueberries or other blueberry varieties. The scant existing data regarding appetite regulation points toward the potential for blueberries to increase the satiety-linked hormone pancreatic polypeptide in humans [1] and to promote satiety in rats [2]. Furthermore, an afternoon snack of berries, which included 25% blueberries by weight, acutely decreased food intake 16% more than a sugar-sweetened refined snack in 12 post-menopausal unrestrained eaters despite no apparent effect on subject ratings of appetite [3].

Blueberries provide fiber, vitamins, minerals and numerous bioactives such as anthocyanins and polyphenols, phenolic acids, organic acids, and other potentially bioactive compounds that have been linked to health benefits. Molan et al. [2] suggested that the capacity of a water-soluble blueberry extract to reduce food intake and lower body weight in rats may be due to one or more of these compounds. More recently, research on wild blueberries has demonstrated the capacity of 1 cup of whole blueberries to elevate plasma concentrations of pancreatic polypeptide in adult humans, although no differences for subjective ratings of appetite were detected [1]. Consistent with the suggestion that blueberry bioactives may regulate appetite, food intake, and body weight, studies on other polyphenol-rich foods and products such as grape juice, apple juice extracts, and dark chocolate have demonstrated potential effects that could promote weight control [4], [5], [6], [7]. Interestingly, of all fruits, increased consumption of blueberries has been most strongly linked to loss of body weight [8]; therefore, exploring this potential in a controlled intervention is essential.

Beyond their effects on appetite and weight regulation, blueberries also positively influence key cardiometabolic biomarkers including improved lipid metabolism, enhanced insulin sensitivity, improved blood pressure and endothelial function, and reductions in inflammatory mediators such as C-reactive protein (CRP) [9]. Animal investigations point toward bioactive blueberry compounds exerting their effects through activation of AMP-activated protein kinase (AMPK) and peroxisome proliferator-activated receptor (PPAR) pathways [10], [11]. Numerous studies in rodent models strongly support the concept that blueberries possess the capacity to improve metabolic processes linked to disease risk [10], [12], [13], [14], [15], [16], [17]. These outcomes are supported by an array of human clinical trials that have also demonstrated improvements in insulin resistance and glucose metabolism after blueberry consumption [18], [19], [20], [21]. Longer interventions (6 months in duration) have demonstrated increases in circulating concentrations of HDL-cholesterol and reductions in triglycerides and CRP that are sometimes not evident after short-term (8 weeks) supplementation [22], [23].

Improved metabolic health as a result of blueberry consumption is relevant to weight control because blood glucose concentrations are known to influence cardiovascular risk as well as appetite responses [24], since meal-dependent fluctuations of blood glucose and corresponding endocrine signals such as insulin are thought to provide important regulatory input for central nervous processing of hunger and satiety. More stable blood glucose concentrations are associated with reduced hunger sensations [25], and acutely elevated insulin concentrations typically increase ratings of fullness and/or limit energy intake [26], [27].

Since obesity has been linked to cardiometabolic abnormalities and appetitive dysregulation [28], identification of foods that impact appetite, body weight, and cardiometabolic risk factors can have significant public health outcomes. Our primary goals were to investigate the potential for intact wild blueberries to serve as an adjunct treatment for weight loss and cardiometabolic responses and appetite-related mechanisms. We hypothesized that daily consumption of 1 cup of whole wild blueberries provided in a yogurt matrix would promote positive appetitive responses via appetite-linked hormones and would enhance weight loss and cardiometabolic responses in comparison to a lower volume, highly processed blueberry-flavored syrup.

## 2 Methods

### 2.1 Participants

Thirty-eight overweight or obese (BMI: 25-40 kg/m^2^) men and women aged 18-50 years were recruited for this study. BMIs of men (29.8 ± 3.5 kg/m^2^) and women (31.4 ± 5.6 kg/m^2^) were similar (p > 0.05) overall and within groups for men (blueberry: 29.0 ± 30.6 kg; syrup: 30.4 ± 3.8 kg) and women (blueberry: 31.0 ± 4.5 kg; syrup: 31.8 ± 4.8 kg). Although no data are available to demonstrate the sample size needed to detect an influence of blueberries on promotion of weight loss, our *a priori* sample size estimation was based on research of other fruits using a similar approach suggests that a minimum of 12 and 6 subjects are needed to detect added weight loss effects for apples and pears, respectively [29].

Participant exclusion criteria included: pregnancy, smoking, greater than 5 kg weight fluctuation in the last 3 months, exercising more than two times per week, medical conditions or use of medications or supplements that might affect body weight or other outcome variables, aversion or allergy to blueberries or yogurt, already eating blueberries more than twice weekly, and consumption of greater than 50 g per day of alcohol. Subjects were recruited by flyers posted on the San Diego State University (SDSU) campus and on social media platforms such as LinkedIn and Instagram. This study was approved by the Institutional Review Board of San Diego State University. Informed consent was obtained from all individual participants included in the study.

### 2.2 Study design

The study utilized a parallel-arm, randomized, single-blind design. Although participants were aware of whether they were consuming blueberries or blueberry-flavored syrup, during recruitment they were informed that they would be consuming a “blueberry yogurt parfait” but were not made aware that a different group was eating a different food mixed with yogurt. Investigators who interacted with participants were unaware of group assignments. Following recruitment, subjects were randomly assigned into two groups, which were stratified for BMI and sex. The blueberry group consumed 1 cup frozen wild blueberries (*Vaccinium angustifolium*) mixed with ¾ cup low-fat yogurt (Mountain High brand) and 1 Equal sweetener packet used to produce similar sweetness to the placebo. The total energy content was estimated to be 170 kcal. Comprehensive data on the composition of wild blueberries have been published elsewhere [30], [31], [32], [33] and indicate that blueberries are rich in polyphenols and other bioactive constituents. One cup of wild blueberries contains approximately 80 kcal, 0.3 g fat, 20 g carbohydrate (14 g sugar), 6 g fiber, and 0.5 g protein. The control group (syrup group) consumed an isocaloric feeding of ¾ cup low-fat yogurt mixed with 2 tablespoons of sugar-sweetened, artificially flavored and colored blueberry syrup (Torani brand). According to the food label, this product contains cane sugar, water, natural flavors, citric acid, sodium benzoate, potassium sorbate, FD&C Red #40, and FD&C Blue #1, and 2 tablespoons provide 80 kcal, 0 g fat, 19 g carbohydrate (19 g sugar), 0 g fiber, and 0 g protein. Bioactive compounds were not assessed for wild blueberries, syrup or yogurt for this study. All yogurt-based snacks were provided to participants in frozen containers along with instructions to allow them to thaw overnight in the refrigerator. Participants were asked to return all uneaten yogurt parfaits to the laboratory when visiting the laboratory to obtain their new periodic supply of parfaits and at the end of the 8-week intervention. Notably, freezing blueberries has been demonstrated to promote nutrient retention [34].

Participants were required to attend three laboratory visits on the SDSU campus over a span of approximately 9 weeks. During an initial laboratory visit, participants were instructed to sign the consent form and usual physical activity was assessed. Participants were then instructed for at home daily weighing. Investigators also instructed participants on the use of the MyFitnessPal app to track their daily dietary intake for the duration of the study. The app was used as a behavioral tool to help promote restriction of food intake. Additionally, to control for testing variability for the next morning, participants were given a standardized 790 kcal meal (Stouffer's brand frozen French bread cheese pizza) to consume for dinner the night before the baseline laboratory testing visit. The baseline visit also included assessment of acute post-prandial responses to consumption of their respective yogurt dish. Participants received text reminders to consume the standardized meal as the last food consumed for the day and then to fast for 12 h prior to their scheduled visit time.

Baseline testing prior to the acute appetite testing session included assessing cravings over the past 7 days, collection of anthropometric data including height, weight, body composition, and waist circumference, and blood pressure. Venous blood was then collected in vacuum containers containing clot activator and gel separator for serum and in vacuum tubes containing EDTA for plasma. Tubes were centrifuged at 1200 X g for 10 min at 4 degrees C.

A 2-h acute postprandial feeding trial was initiated beginning with collection of arterialized fingerstick blood samples into Greiner Bio-One MiniCollect™ Capillary Blood Collection System Tubes containing EDTA. Participants were then provided their assigned feeding to consume within 5 min. Timing was set at 5 min to standardize the effect of appetitive response to food intake across all participants. Arterialized blood was again collected 30, 60 and 120 min after food intake for analysis of plasma concentrations of glucose, insulin, ghrelin, and pancreatic peptide. Appetitive responses (hunger, fullness, desire to eat, and prospective food consumption) were assessed by 100-mm visual analog scales at baseline and 15, 30, 45, 60, 90, and 120 min after intake.

During the subsequent 8 weeks, both groups were counseled for weight loss. This was achieved through a comprehensive program including a) dietary counseling, b) daily text messaging, and c) daily at-home weighing with a Wi-Fi-enabled scale. Participants were informed to consume the 170-kcal yogurt parfait to which they were assigned (containing either wild blueberries or placebo syrup) as a mid-afternoon snack daily. Mid-afternoon snacks were chosen with the goal of reducing food intake for the remainder of the day. The participants met with investigators every two weeks for intensive virtual weight-loss counseling to ensure adherence to the dietary prescription and provide participants with tips to practice in daily life to achieve effective weight loss. The dietary prescription consisted of a 500 kcal/day goal energy reduction from baseline levels determined using MyFitnessPal. Participants were instructed to maintain usual physical activity. The final visit occurred at the end of the 8-week intervention. Participants completed physical activity and cravings questionnaires, final assessments of anthropometric and blood pressure measurements, and a venous blood draw.

### 2.3 Procedures

Blood pressure was assessed in triplicate using an automated cuff (M3, Omron Healthcare, Inc; Kyoto, Japan) with participants in a comfortable, seated position with both feet on the floor. Both arms were tested for blood pressure, and the arm exhibiting the higher reading was used for all testing. Body composition was assessed using multi-segmental, multi-frequency bioelectrical impedance analysis (InBody 770, Biospace Inc; Cerritos, CA). Physical activity for the preceding month was assessed using the Global Physical Activity Questionnaire (WHO; Geneva, Switzerland), which assessed metabolic equivalents for work, transportation, recreating and sedentary activities. Food cravings over the prior 7 days were assessed using the Control of Eating Questionnaire.

Daily weights were collected with a Withings Body + Smart Scale (UK). Participants were instructed to download the Withings app on a smartphone or tablet, which provided data directly to investigators each day in order for researchers to provide weight loss encouragement if necessary. Weights were collected each morning upon awakening, after voiding their bladder, and without clothing.

The daily text messages sent were consistent for every participant. Each individual received the same text message on the same days throughout the 8-week intervention. The text messages were organized by different categories and sent on a rotation based on the day of the week. This design was inspired by a previous study that utilized daily text messages as a weight-loss intervention [35].

Plasma glucose was assayed using a commercially available colorimetric kit (EKF Diagnostics, Boerne TX). Insulin and CRP were assessed using ELISA kits (ALPCO, Salem NH). Ghrelin was assessed using an ELISA kit from Raybiotech (Peachtree Corners, GA), and pancreatic polypeptide was measured with an ELISA kit from Invitrogen (Carlsbad, CA). Blood lipid profiles (total cholesterol, HDL-cholesterol and triglycerides) were assessed using colorimetric kits from EKF Diagnostics (Boerne, TX). LDL-C was determined using the equation of Friedewald et al. [36].

### 2.4 Statistical analyses

Data were statistically analyzed using JASP version 0.16.2 (Amsterdam, Netherlands). Descriptive data collected were analyzed using independent T-tests for differences between groups. All data were evaluated for normality using the Shapiro-Wilk test; non-normally distributed data were logarithmically transformed prior to analysis. However, if data continued to be non-normally distributed, nonparametric Friedman's Test and Mann-Whitney Test were used. Friedman's Test was used to evaluate within group changes over time for all appetite hormones. The Mann-Whitney test was used to evaluate differences between groups for appetite hormones at all time points. Any normally distributed data were analyzed parametrically using repeated measures ANOVA and paired T-tests within trials and independent T-tests between groups when warranted as post-hoc analyses. Statistical significance was accepted at a p-value ≤ 0.05.

## 3 Results

### 3.1 Study participants

A total of 105 individuals were screened for eligibility; 67 were excluded for not meeting inclusion criteria. The remaining 38 participants (11 male and 27 female), aged 19 to 49 years (mean age 26.2 ± 8.7 years), (mean BMI 30.9 ± 4.3 kg/m^2^; men: 29.8 ± 3.5; women: 31.4.± 4.5) were enrolled. No statistically significant differences between groups were detected. Of the 38 participants enrolled, 36 completed the study, and 2 were withdrawn due to noncompliance with the study protocol. All analyzed participants reported 100 percent compliance to consuming the yogurt parfait interventions. A complete participant flow diagram can be found in Fig. 1.

**Fig. 1:**
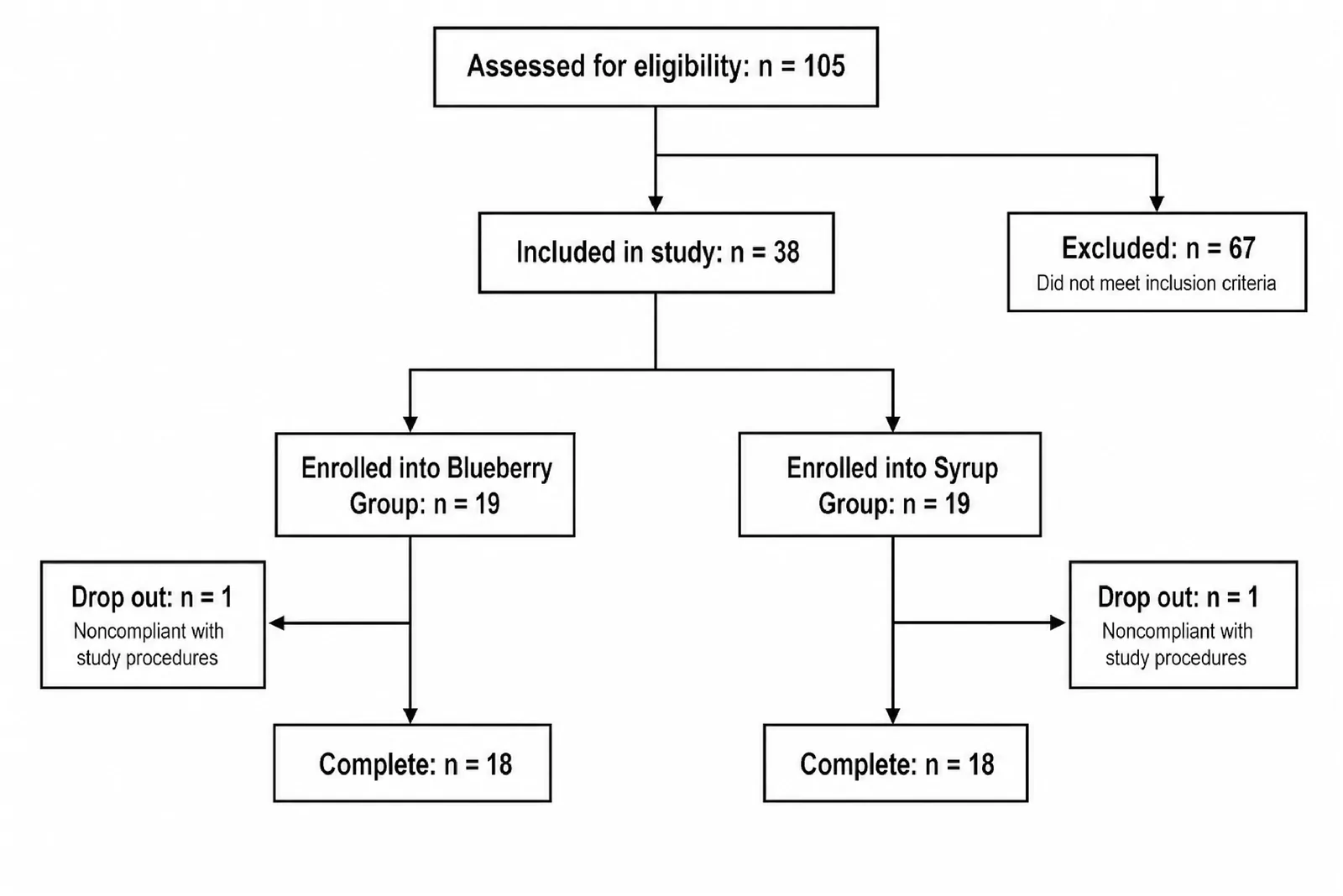
Participant flow diagram.

### 3.2 Anthropometrics & blood pressure

Both interventions resulted in similar weight loss (Table 1). The weight loss difference was 0.09 ± 2.4 kg (95% CI [−1.00, 1.17]) for wild blueberries in comparison to syrup. No additional benefit of either blueberries or artificially flavored and sweetened syrup was detected, although extremely modest effects for enhanced effects of artificially flavored and sweetened syrup cannot be excluded. No significant differences between wild blueberry and syrup groups for changes for anthropometric or body composition measures were detected. Percent weight change was −1.32 ± 2.26% for the wild blueberry group and −1.55 ± 1.58% for the syrup group. Lean body mass and body fat mass both decreased significantly over time; however, post-hoc analysis revealed that the changes were statistically significant within the syrup group only (p = 0.008 and p = 0.002 for lean body mass and body fat mass, respectively). No changes in systolic or diastolic blood pressures were detected for either group.

**Table 1:** Anthropometrics and blood pressure responses for participants consuming blueberries or blueberry-flavored syrup.

*Variable*	*Blueberry Baseline*	*Blueberry Final*	*Syrup Baseline*	*Syrup Final*	*P (Time × Treatment)*	*P (Time)*	*P (Treatment)*
*Weight (kg)*	83.1 ± 12.3	82.0 ± 12.3*	88.2 ± 16.2	86.9 ± 16.6*	0.869	**<0.001**	0.313
*Waist Circumference (cm)*	88.7 ± 10.6	85.5 ± 11.0	92.8 ± 12.5	92.0 ± 13.7	0.353	0.105	0.182
*PBF (%)*	39.7 ± 7.9	39.3 ± 8.6	37.3 ± 8.4	36.9 ± 8.6	0.932	0.284	0.416
*LBM (kg)*	49.8 ± 8.1	49.4 ± 8.6	54.1 ± 11.5	53.6 ± 11.7*	0.953	**0.036**	0.227
*SMM (kg)*	27.8 ± 4.9	27.6 ± 5.3	30.3 ± 6.9	30.1 ± 7.1	0.980	0.074	0.244
*BFM (kg)*	33.3 ± 9.7	32.9 ± 10.5	32.3 ± 9.3	31.5 ± 9.4*	0.812	**0.039**	0.750
*TBW (L)*	36.5 ± 5.8	36.2 ± 6.2	39.6 ± 8.4	39.3 ± 8.6*	0.875	**0.031**	0.226
*Systolic BP (mmHg)*	111.1 ± 9.6	110.1 ± 9.8	114.7 ± 14.6	117.9 ± 12.6	0.110	0.412	0.146
*Diastolic BP (mmHg)*	70.6 ± 7.1	70.4 ± 7.1	73.1 ± 11.3	73.5 ± 8.9	0.794	0.949	0.308

Values are represented as mean ± standard deviation.

P value for repeated measures ANOVA.

* Indicates a significant difference (P < 0.05) within a trial from baseline to final.

PBF = percent body fat; LBM = lean body mass; SMM = skeletal muscle mass; BFM = body fat mass; TBW = total body water; BP = blood pressure.

### 3.3 Cravings & physical activity

A significant main effect of time was observed for increased cravings control during the weight loss interventions (p = 0.044) and tendency (p = 0.059) was detected for a time by treatment interaction (Table 2). Post-hoc analysis revealed that this reduction was confined to the blueberry group only (p = 0.006). Significant main effects of time for decreases in cravings for both sweet (p = 0.002) and savory foods (p = 0.008) were also observed, which were statistically significant within both groups but not different between groups. No significant changes were observed for feelings of hunger or fullness for the 7-day questionnaire period. No significant changes in overall or subcategories of physical activity level were observed during the intervention (Table 3).

**Table 2:** Seven-day craving and appetite responses for participants consuming blueberries or blueberry-flavored syrup.

*Variable*	*Blueberry Baseline*	*Blueberry Final*	*Syrup Baseline*	*Syrup Final*	*P (Time × Treatment)*	*P (Time)*	*P (Treatment)*
*Cravings Control*	46.5 ± 18.1	60.9 ± 12.7*	54.6 ± 18.2	55.1 ± 14.6	0.059	**0.044**	0.776
*Cravings for Sweet*	42.6 ± 22.9	25.5 ± 18.9*	36.2 ± 22.9	27.6 ± 18.9*	0.275	**0.002**	0.695
*Cravings for Savory*	45.4 ± 21.8	34.7 ± 23.5*	43.1 ± 18.6	35.6 ± 21.8*	0.622	**0.008**	0.908
*Hunger*	42.9 ± 16.0	30.1 ± 15.2	42.0 ± 19.7	39.2 ± 17.5	0.287	0.098	0.276
*Fullness*	58.8 ± 14.5	46.8 ± 21.6	50.5 ± 16.1	50.9 ± 17.4	0.104	0.128	0.668

Values are represented as mean ± standard deviation.

P value for repeated measures ANOVA.

* Indicates a significant difference (P < 0.05) within a trial from baseline to final.

**Table 3:** Physical activity responses for participants consuming blueberries or blueberry-flavored syrup.

*Variable*	*Blueberry Baseline*	*Blueberry Final*	*Syrup Baseline*	*Syrup Final*	*P (Time × Treatment)*	*P (Time)*	*P (Treatment)*
*Work METS*	13.8 ± 20.8	25.5 ± 39.7	14.0 ± 22.2	13.5 ± 18.9	0.242	0.280	0.453
*Transport METS*	16.3 ± 31.5	14.7 ± 17.6	13.2 ± 16.5	5.5 ± 8.8	0.466	0.274	0.287
*Recreation METS*	27.5 ± 65.2	17.2 ± 26.9	5.1 ± 9.3	13.6 ± 16.8	0.290	0.918	0.162
*Sedentary METS*	7.1 ± 2.5	7.5 ± 3.1	5.9 ± 2.9	7.1 ± 2.5	0.335	0.088	0.348

Values are represented as mean ± standard deviation.

P value for repeated measures ANOVA.

### 3.4 Cardiometabolic biomarkers

Baseline biomarker values did not differ significantly between groups (Table 4). No statistically significant main effects or interactions were observed for any biomarker; however, insulin strongly tended (p = 0.055) to demonstrate a main effect of time for a decrease in concentration.

**Table 4:** Cardiometabolic biomarker responses for participants consuming blueberries or blueberry-flavored syrup.

*Measurement*	*Blueberry Baseline*	*Blueberry Final*	*Syrup Baseline*	*Syrup Final*	*P (Time X Treatment)*	*P (Time)*	*P (Treatment)*
*Blood Glucose (mg/dL)*	97.7 ± 8.5	98.9 ± 7.1	98.1 ± 11.6	100.9 ± 13.0	0.768	0.198	0.585
*Insulin (mIU/mL)*	13.5 ± 4.5	12.1 ± 4.9	15.4 ± 7.7	14.0 ± 7.4	0.345	0.055	0.392
*CRP (mg/L)*	1.05 ± 0.96	0.95 ± 1.02	1.28 ± 1.21	1.15 ± 1.12	0.574	0.121	0.590
*HDL-C (mg/dL)*	68.0 ± 19.8	69.4 ± 18.5	65.2 ± 22.9	64.2 ± 23.6	0.532	0.494	0.480
*LDL-C (mg/dL)*	72.5 ± 19.2	74.3 ± 18.3	70.7 ± 24.4	69.6 ± 27.9	0.657	0.726	0.563
*Total Cholesterol (mg/dL)*	148.9 ± 18.3	152.9 ± 21.3	144.4 ± 20.2	142.2 ± 20.6	0.199	0.988	0.139
*Triglycerides (mg/dL)*	41.8 ± 14.1	46.3 ± 18.7	41.9 ± 13.3	41.7 ± 16.3	0.697	0.494	0.448

Values are represented as mean ± standard deviation.

P value for repeated measures ANOVA.

Abbreviations: CRP, C-Reactive Protein; HDL-C, High-density lipoprotein cholesterol; LDL-C, Low-density lipoprotein cholesterol.

### 3.5 Acute appetitive responses

Hunger, fullness, desire to eat, and prospective food consumption ratings did not differ significantly between the blueberry and syrup groups during the acute feeding trials (Fig. 2). However, all four measures demonstrated a significant main effect of time (p < 0.001). Hunger decreased and fullness increased initially after consumption and returned toward baseline thereafter. Both desire to eat and the desired amount to eat decreased postprandially and gradually increased until 120 min. Changes in plasma concentrations of ghrelin, pancreatic polypeptide, insulin, and glucose were largely similar between blueberry and syrup groups, except that ghrelin was transiently lower for the syrup group at the 30-min timepoint despite no main effects or interaction (Fig. 3). Insulin and glucose both demonstrated significant main effects of time, with both groups’ concentrations rising postprandially and declining steadily between 30 and 120 min (p < 0.001). No main effects or interaction were detected for pancreatic polypeptide.

**Fig. 2:**
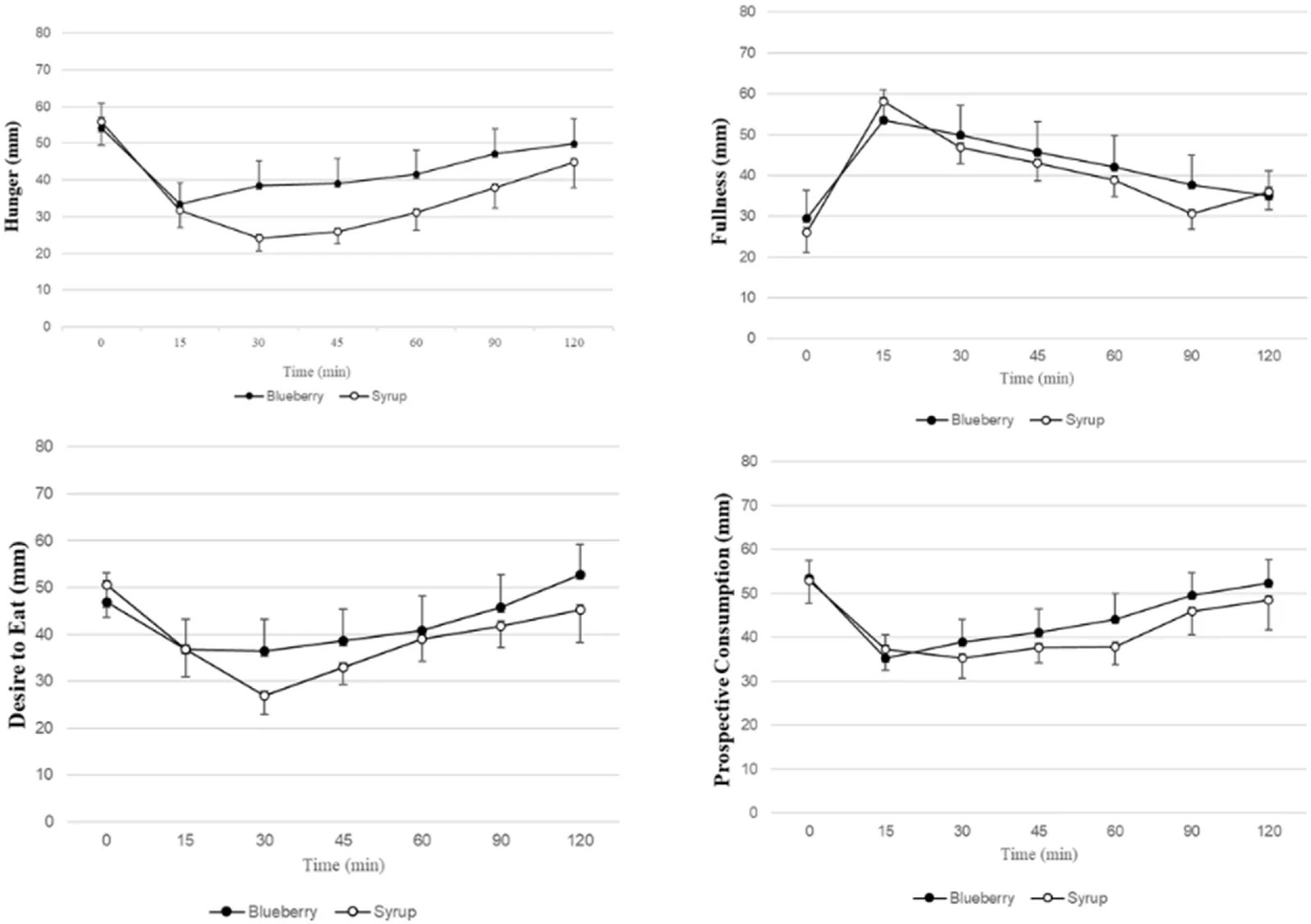
Perceived appetite response after consumption of blueberries (•) or blueberry-flavored syrup (໐) yogurt cups. Data are presented as means ± SE, n = 38. Within trials, all time points were significantly different from baseline, p < 0.05. For hunger, fullness, desire to eat, and prospective consumption, there were no significant main effects or interactions between responses from blueberries or syrup.

**Fig. 3:**
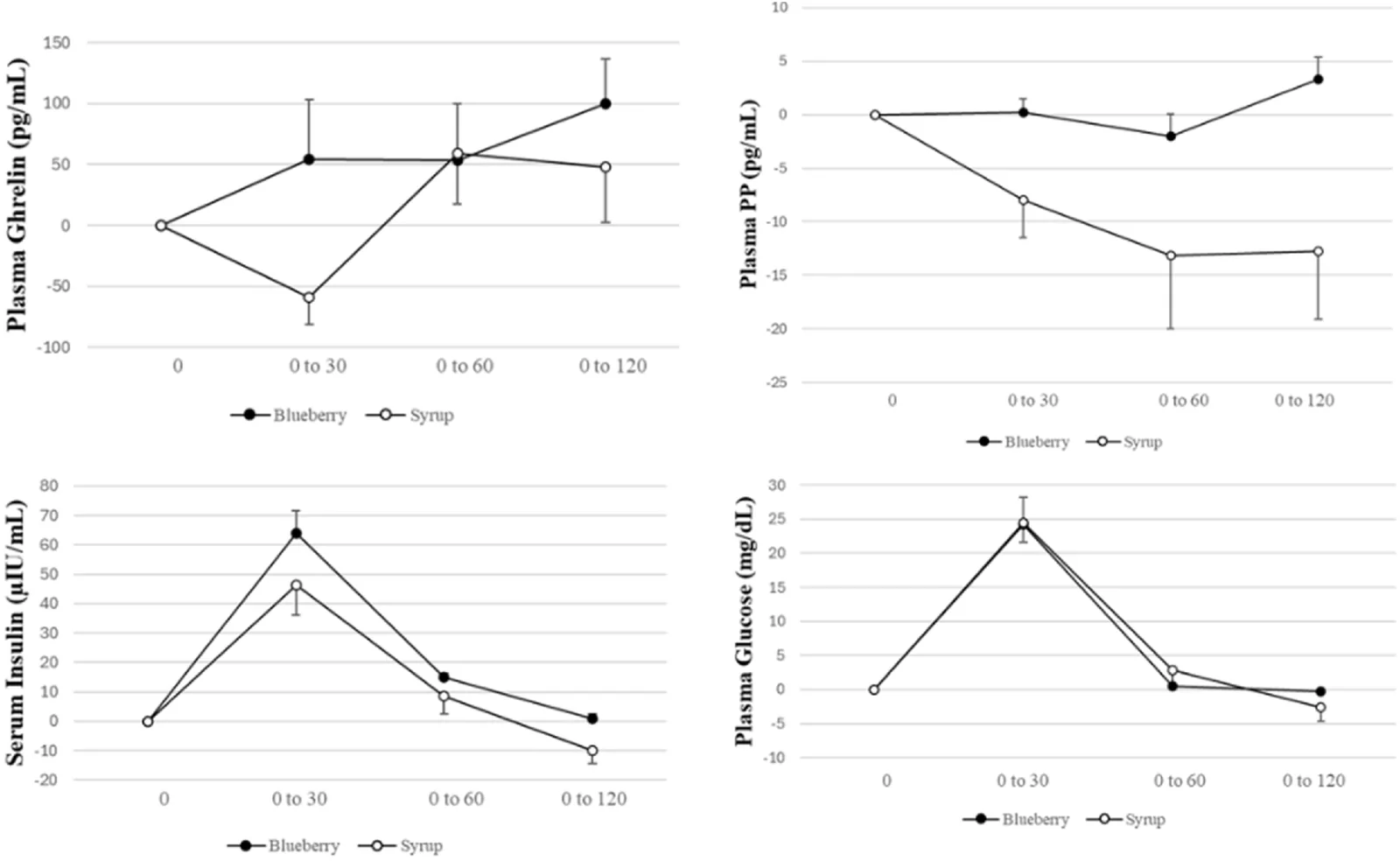
Appetitive hormone and glucose responses after consumption of blueberries (•) or blueberry-flavored syrup (໐) yogurt cups. Data are presented as means ± SE, n = 38. Glucose and insulin were significantly different from baseline at minute 30, p < 0.05. Ghrelin was significantly different from baseline at minute 30 for the syrup group but not blueberry group. There were no significant differences between responses from the blueberry or syrup groups for all other responses.

## 4 Discussion

Contrary to our hypotheses, consumption of whole, intact wild blueberries within a yogurt-based snack did not impact acute appetitive responses or longer-term weight loss and cardiometabolic outcomes compared to a sugar-sweetened control yogurt snack during 8 weeks of energy restriction despite improving cravings control. However, since participants successfully lost minor amounts of weight during either blueberry or syrup consumption, despite being asked to add 170 kcal daily yogurt-based snacks to their diets, it is possible that potential differential influences of whole wild blueberries versus a highly processed, sugar-sweetened, artificially flavored blueberry syrup were masked by the yogurt component of the feedings. Since dietary intake records were unavailable to the investigators and mean weight loss was modest, the actual energy deficit achieved by participants could not be verified.

Although post-hoc analyses of the main effect of time for lean mass loss revealed statistically significant changes within the syrup group only, the potential preservation of lean mass observed within the wild blueberry group should be interpreted carefully. Furthermore, within group analysis also revealed no change in fat mass within that trial despite overall weight loss. These results should be interpreted with a high degree of caution to avoid differences in nominal significance (DINS) error. As recently reviewed, anthocyanins such as those provided by blueberries may exert impacts on muscle protein synthesis; however, such effects may require a simultaneous exercise intervention, which did not occur in this study [37].

Despite the availability of limited evidence suggesting that blueberries or blueberry extract may influence satiety-linked hormones, such as pancreatic polypeptide [1], and reduce food intake in animal models [2], no major treatment effects were observed in our study for differences in postprandial appetite hormone responses or subjective appetite measures. In support of these effects, no differences in postprandial glucose and insulin responses between the whole blueberry and refined blueberry syrup trials, which is surprising given the differences in the degree of refinement, nutrition compositions and the complexities of the food matrices. However, despite having a relatively low glycemic index [38], past research has also demonstrated that in comparison to a sugar-matched control, freeze-dried blueberry powder failed to reduce blood glucose excursions of individuals with type 2 diabetes [21]. Other researchers have demonstrated the capacity for whole blueberries to robustly lower the glycemic response when consumed along with white bread [39], which occurred with a modestly higher insulinemic response. Discrepancies between findings may relate to differences in experimental design, anthocyanin dose, or the influence of the food matrix on bioavailability. More research is necessary to draw meaningful conclusions.

As stated previously, the yogurt matrix used in this study may have contributed to the absence of treatment differences. Dairy proteins and organic acids can attenuate postprandial glycemic responses by slowing gastric emptying and stimulating insulin secretion [40], [41], which may have masked potential blueberry-specific effects. Moreover, although whole blueberries enhance texture and impart greater volume than highly processed syrup, both of which have been linked to enhanced satiety through increased mastication and reduced energy density [3], [42], these effects were not evident from either our acute or longer-term data. Again, provision of the intervention foods within a yogurt matrix may have masked the impacts of whole, intact wild blueberries versus the highly processed syrup. Therefore, future studies should assess the impacts of wild blueberries versus an isoenergetic control food such as a blueberry-flavored, sugar-sweetened syrup on similar outcomes without the inclusion of yogurt as a vehicle to provide the feedings, although inability to provide the interventions in a blinded fashion would confound the results. The outcomes of this study highlight some of the challenges of studying the influence of specific foods on health-related variables.

Behavioral components of the intervention likely played a significant role in the overall weight loss achieved by both groups. Participants received individualized counseling, daily text messages, and remote self-monitoring, all of which are known to promote adherence to study protocols and weight reduction [43], [44]. These evidence-based behavioral strategies may have overshadowed any modest physiological effects of bioactive components or physical properties of wild blueberries. Given that snacks contribute approximately one-quarter of daily energy intake in U.S. adults [45], [46], replacing typical energy-dense snacks with a nutrient-rich blueberry yogurt parfait could serve as a sustainable and healthful dietary behavior [18], [19] regardless of its capacity to differentially influence body weight.

Although the present study did not demonstrate statistically significant effects of long-term wild blueberry consumption on cardiometabolic outcomes including blood pressure and circulating concentrations of glucose, insulin, blood lipids or CRP, the participants exhibited very healthy profiles for these variables at baseline. This likely limited the potential for wild blueberries to yield improvements. Previous trials extending beyond 12 weeks have demonstrated more pronounced changes in lipid profiles and inflammatory markers, indicating that the duration of exposure may be a key determinant of measurable metabolic benefit [22], [23]. Notably, however, participants in those studies exhibited underlying metabolic health conditions (i.e., metabolic syndrome [22] and type 2 diabetes mellitus [23]. Mechanistically, anthocyanin-rich foods such as blueberries have been shown to modulate lipid and glucose metabolism through activation of AMPK and PPAR signaling, which enhances fatty acid oxidation, improves insulin sensitivity, and attenuates NF-KB-mediated inflammatory pathways [10], [11], [47]. Within our study, the tendency for decreasing plasma insulin concentration may have been secondary to the weight loss intervention or the yogurt-based feeding rather than a specific effect of blueberries since the effect tended to occur similarly for both blueberries and blueberry syrup.

Overall, this study suggests that inclusion of either wild blueberries or blueberry syrup within a yogurt matrix as a daily snack can fit into a weight loss regimen, although weight loss was not particularly robust. Notably, the metabolic effects of wild blueberries may be limited in this relatively young and healthy population and/or their effects may have been masked by the yogurt-based food matrix and/or behavioral components of the weight loss intervention. Findings from both the acute and long-term trials indicate that wild blueberry consumption largely did not alter short-term appetite regulation or longer-term weight loss, lipid profiles or inflammation status relative to a highly processed isocaloric control syrup when provided within a yogurt matrix in generally healthy adults. Future studies should assess whether whole wild blueberries impact metabolic and appetitive responses when fed without yogurt, which may have masked some key outcomes.

## 5 Conclusion

Despite improving control of food cravings, when consumed within a yogurt-based food matrix, consumption of whole, intact wild blueberries did not enhance weight loss or improve cardiometabolic responses during structured energy restriction. Consistent with the long-term outcomes, acute appetitive responses to whole wild blueberries were similar to the highly processed control syrup used to flavor and sweeten the yogurt. Nevertheless, the potential for wild blueberries to curb food cravings and to provide a nutrient-dense alternative to typical snack foods represents meaningful findings with practical dietary implications. Given their favorable nutrient profile and polyphenol content, wild blueberries can be feasibly included within comprehensive weight management interventions. Future investigations should expand upon these findings by examining dose-response relationships, exploring alternative delivery matrices, and assessing longer-term effects on metabolic health and body composition.

## CRediT authorship contribution statement

**Mark Kern:** Writing – review & editing, Writing – original draft, Supervision, Project administration, Methodology, Investigation, Funding acquisition, Formal analysis, Conceptualization. **Parker Contreras:** Writing – original draft, Formal analysis, Data curation. **Emily Hickey:** Writing – original draft, Formal analysis, Data curation. **Sarah Parsons Lee:** Writing – original draft, Visualization, Formal analysis, Data curation. **Jenna Mueller:** Formal analysis, Data curation. **Shirin Hooshmand:** Writing – review & editing, Visualization, Supervision, Investigation, Funding acquisition, Formal analysis, Conceptualization.

## Funding

This work was supported by the Wild Blueberry Association of North America. (Grant no. S00029660)
